# Lactic acidosis and hypoglycemia as markers of disease progression of multiple myeloma: A case report

**DOI:** 10.1002/jha2.176

**Published:** 2021-05-05

**Authors:** Caroline Ziegler, Lev Volkov, Rémy Marnai, Guilhem Courte, Aurélie Cravoisy, Marie Conrad, Lionel Nace, Agnès Melone, Aurore Perrot, Sébastien Gibot

**Affiliations:** ^1^ Nancy Regional and University Hospital Center (CHRU) Nancy France

**Keywords:** acidosis, hypoglycemia, lactic, multiple myeloma

## Abstract

**Case report**: A 64‐year‐old man was hospitalized in the intensive care unit with pneumonia, lactic acidosis, and hypoglycemia. Investigations revealed a kappa light chain multiple myeloma. The patient underwent chemotherapy by bortezomib, lenalidomide, and dexamethasone. Serum lactate level and glycemia normalized. Evaluation at day 28 showed a disease progression. Lenalidomide was switched for daratumumab, bortezomib, and dexamethasone. In front of the inefficiency of the chemotherapy, the patient underwent third‐line chemotherapy by melphalan. There was a correlation between the evolution of the myeloma, serum lactate level, and hypoglycemia, with a normalization after chemotherapy, and a rise at myeloma's relapse. Daratumumab was continued as a maintenance treatment. The patient died 4 months and 10 days after his first hospital admission.

**Discussion**: Our case is consistent with a type B tumor‐associated aerobic glycolytic lactic acidosis, called the Warburg effect. It is well described in association with other hematologic malignancies, but rarely in association with myeloma. All reported cases of myeloma with type B lactic acidosis died within 1 year.

**Conclusion**: When associated with multiple myeloma, tumor‐associated aerobic glycolytic lactic acidosis is correlated with the disease progression and has a very high mortality rate.

***Significance Statement*** : Aerobic glycolytic lactic acidosis also known as the Warburg effect can be encountered in multiple myeloma, resulting of a metabolic shift to increased glycolysis operating in malignant cells. Together with hypoglycemia, it is well correlated with the disease progression and has a very poor outcome.

## 1 INTRODUCTION

Multiple myeloma is a clonal plasma cell proliferative disorder. Malignant plasma cells invade the bone marrow and overproduce abnormal immunoglobulin or light chain protein, causing fatigue, weight loss, immunodepression, bone pain, hyperviscosity, anemia, hypercalcemia, and renal insufficiency. Multiple myeloma is the second most common hematologic malignancy and accounts for less than 2% of all cancers, with a median presentation age of 70 years [1, 2, 3]. The improvement of multiple myeloma treatment has globally improved its mortality, with an overall 5‐year survival rate of 52% [4].

A wide variety of metabolic disorders are observed in patients presenting hematological malignancies, such as hyperuricemia, hypercalcemia, hypo‐ or hyperglycemia, lactic acidosis, and inappropriate secretion of antidiuretic hormone syndrome [5]. Pathogenetic mechanisms of these metabolic abnormalities are extremely various, including neoplastic cell turnover, tumor secretion factors, auto‐immune reactions, and can also be treatment‐induced [5].

We describe a case of multiple myeloma associated with high and recurrent hyperlactatemia and profound hypoglycemia.

## 2 CASE REPORT

A 64‐year‐old man, smoker, without any specific medical history or treatment, was admitted to the emergency department of our hospital in June 2019 with progressive dyspnea, bone pain, and a recent loss of 10 kg.

On examination, blood pressure was normal (126/69 mm Hg), heart rate was raised to 107 beats/min, oxygen saturation was 97% while breathing room air, respiratory rate was raised to 23/min, and the temperature was 37°C. He had clinical signs of dehydration, spontaneous chest and dorsal pain, and vomiting. Laboratory findings showed hemoglobin at 10.5 g/dL (normal rate 13–17 g/dL), thrombocytopenia at 96 G/L (150–450 G/L), white blood cell count at 8.4 G/L (4–10 G/L), lymphopenia at 0.64 G/L (1–4 G/L), creatinine at 42 micromol/L (62–115 micromol/L), BUN at 5.9 mmol/L (3.8–8.2 mmol/L), natremia at 131 mmol/L (136–145 mmol/L), hypokalemia at 2.97 mmol/L (3.4–4.5 mmol/L), hypercalcemia at 3.59 mmol/L (2.18–2.6 mmol/L), bicarbonates at 15.7 mmol/L (22–28 mmol/L), hypophosphatemia at 0.69 mmol/L (0.78–1.65 mmol/L), hypoprotidemia at 42 g/L (57–82 g/L) with hypoalbuminemia at 26.8 g/L (32–46 g/L), ASAT at 44 U/L (13–40 U/L), ALAT at 30 U/L (7–40 U/L), and total bilirubin concentration at 11 micromol/L (3–19 micromol/L). computed tomography (CT) scan showed bilateral pneumonitis, multiple bone lesions of the spine, sternum and ribs, and no pulmonary embolism. The cardiac echography was normal. The patient received bisphosphonate and intra‐venous hyperhydration. On the next day, his respiratory condition worsened. The oxygen saturation rate lowered, and high‐flow nasal cannula oxygen therapy with FiO2 60% was initiated. Arterial blood gas showed respiratory alkalosis and lactic acidosis: pH 7.46, PCO2 23 mm Hg, PO2 65.2 mm Hg, bicarbonate 16.2 mmol/L, lactate 15 mmol/L. The patient was admitted to the ICU.

**FIGURE 1:**
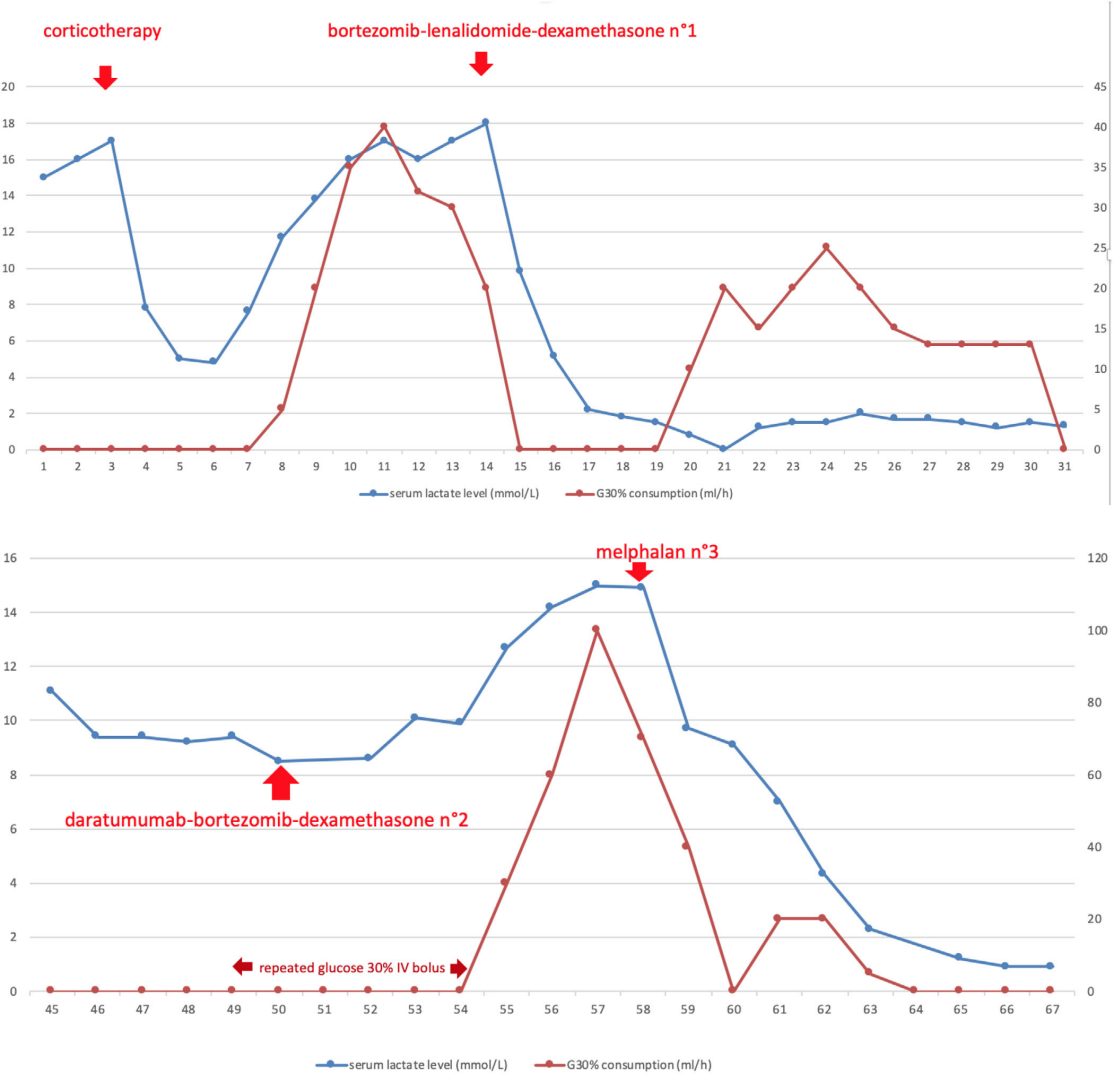
Evolution of serum lactate level (left ordinate) and consumption of glucose 30% (right ordinate) within the days following the initial admission (axis)

Broad‐spectrum antibiotics (cefotaxime and spiramycine) were administered. Three days after the admission in ICU, the patient's condition worsened. A rapidly extensive pulmonary infection was suspected, that required intubation, mechanical ventilation, and norepinephrine infusion. Despite respiratory improvement and hemodynamic stabilization with normal cardiac output, intravenous hydration and thiamine course, serum lactate level remained between 10 and 15 mmol/L. The first attempt of osteomedullary biopsy was not contributive because of a very friable bone. Hypothesizing an aggressive hematological malignancy, intra‐venous corticotherapy of 1 mg/Kg a day was initiated on the 4th day after admission. As a result, tumor lysis syndrome occurred with hypocalcemia of 1.92 mmol/L, hyperphosphatemia of 1.79 mmol/L, hyperuricemia of 475 micromol/L, and acute kidney failure with creatinine of 189 micromol/L and hyperkaliemia of 5.22 mmol/L. Because of kidney failure with anuria, renal replacement therapy was started. In addition, serum lactate level decreased rapidly, although transiently with a new increase up to 15 mmol/L 1 week later, without clinical worsening. Concomitantly to re‐ascension of lactate, a deep hypoglycemia appeared, with an increasing need for glucose 30% infusion to maintain glycemia over 0.9 g/L (Figure 1). Despite very low levels of glycemia, the patient didn't present any neurological glucopenia signs.

**FIGURE 2:**
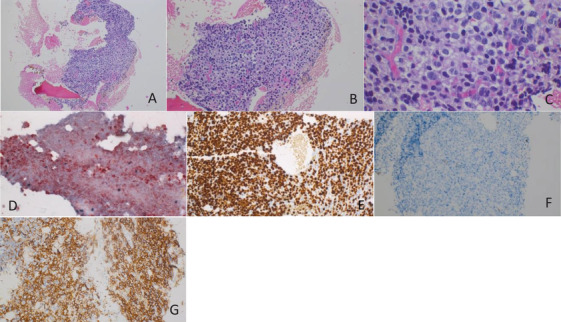
Anatomopathological examination of the osteomedullary biopsy showing a diffuse infiltration by atypical and monotypic plasma cells. (A) Diffuse plasma cell infiltration replacing normal hematopoietic elements (hematoxylin‐eosin, original magnification x10). (B and C) Voluminous plasma cells showing nuclear atypia (hematoxylin‐eosin, original magnification x10 and x60). (D) In situ hybridization lambda/kappa demonstrating kappa monotypic plasma cells. (E) Ki67 proliferative index of 95%. (F and G) Neoplastic plasma cells CD20 negative and CD138 positive

The patient underwent a second, CT‐guided, osteomedullary biopsy of the left wing of the ilium under general anesthesia. Anatomopathological examination showed a massive bone marrow infiltration of atypical plasma cells with a proliferative index of 95% (Figure 2).

In situ hybridization confirmed a monotypic kappa population. Serum electrophoresis and immunofixation showed an oligoclonal profile with minimal monoclonal gammapathies alpha2kappa2, mu2lambda2 and gamma2lambda2, and severe hypogammaglobulinemia at 1.3 g/L. Bence‐Jones proteinuria was 0.76 g/L. Dosage of serum free light chains showed kappa light chains increased to 417.9 mg/L and lambda light chains of 6.4 mg/L, corresponding to an abnormal ratio of 65.19. The final diagnosis was kappa light chain multiple myeloma with four CRAB features (bone lesions, hypercalcemia, renal insufficiency, and anemia), ISS score III. Genetic analysis was not performed.

On the 14th day after admission, the patient received chemotherapy by subcutaneous bortezomib 1.3 mg/m^2^, oral lenalidomide 25 mg once a day 21 d/28, and oral dexamethasone 40 mg. His condition progressively improved, norepinephrine and renal replacement therapy were stopped, and the patient was extubated. Serum lactate level, calcemia, and glycemia normalized, and the light chain ratio lowered to 3.94. However, evaluation at day 28 after the first chemotherapy showed a disease progression with a light chain ratio of 30.2, dFLC (difference of free light chains) of 491.2 and raised serum lactate level (Figure 1).

Lenalidomide was then switched for second‐line chemotherapy with daratumumab 16 mg/kg, bortezomib and dexamethasone. The patient's condition worsened with the occurrence of a ventilator‐associated pneumonia by *Pseudomonas aeruginosa* and *Klebsiella aerogenes* leading to treatment with cefepime, intubation, and then tracheostomy. In front of the inefficiency of the chemotherapy, with a persisting hyperlactatemia, reappearance of hypoglycemia (Figure 1), hypercalcemia, a light chain ratio of 89.14 and dFLC of 553.5, daratumumab and bortezomib were stopped, and the patient underwent third‐line chemotherapy by melphalan 30 mg/m^2^. Again, serum lactate level and light chain ratio decreased (2.72), calcemia, and glycemia normalized. Despite 15 days of aplasia complicated with a *Clostridium difficile* colitis, the patient's condition improved, and the tracheal tube was removed. On the 20th of September, after 3 months in the ICU, the patient was discharged to the hematology ward, with severe amyotrophy, polyneuropathy, and denutrition.

Daratumumab was continued as a maintenance treatment, and the patient was discharged to his home. Unfortunately, he was readmitted 17 days later with a febrile aplasia and bilateral pneumonia. Biological analysis showed a relapse of the myeloma with hypercalcemia of 2.64 mmol/L, a light chain ratio of 52.02, dFLC of 160.7 and raised serum lactate level to 12.8 mmol/L. Understanding his condition, the patient requested palliative care and subsequently died 4 months and 10 days after his first hospital admission.

## 3 DISCUSSION

Our patient presented a very invasive kappa light chain myeloma associated with severe hyperlactatemia and profound hypoglycemia. Serum lactate level decreased after chemotherapy and increased concomitantly with the myeloma relapses.

Lactic acidosis is defined by an elevated blood lactate level, typically >5 mmol/L, depending on the laboratory norm. Acidemia is not constant in lactic acidosis, as coexisting acid‐base disorders can increase the blood pH [6]. Lactic acidosis most often results from an anaerobic glycolytic state related to tissue hypoxia, also called type A lactic acidosis, such as shock, severe hypoxia, organ ischemia, or convulsions. On the other hand, lactic acidosis can also occur in normoxia and is then called type B lactic acidosis. Type B lactic acidosis may arise in case of beta2‐adrenoreceptors stimulation (elevated epinephrine levels, use of beta2‐adrenergic agonists in asthma), in alkalemic disorders (by stimulation of the phosphofructokinase), upon the use of drugs or toxics impairing oxidative phosphorylation (acetaminophen, antiretroviral agents, metformin, propofol), in hyperosmolar hyperglycemic stages, thiamine deficiency, primitive metabolic disorders, decreased lactate clearance (liver failure), and can also occur in cancers and hematological malignancies, involving aerobic glycolysis, then called the Warburg effect [6, 7, 8].

In normal aerobic conditions, glycolysis converts glucose to pyruvate, which is oxidized to acetyl‐CoA, releasing CO2 and entering the Krebs cycle. This reaction produces NADH, which is then used in oxidative phosphorylation and produces 36 molecules of ATP. Under anaerobic conditions, pyruvate is converted to lactate [9]. The Warburg effect is an aerobic glycolytic state where, despite the presence of oxygen, pyruvate is metabolized to lactate producing only two molecules of ATP, appearing to be paradoxically inefficient [10]. However, malignant transformation is linked with changes in the metabolic cellular pathway necessary for cancer cell proliferation, and aerobic glycolysis provides additional substrates, such as amino acids, nucleotides, and lipids, that are not provided if converting all the glucose to CO2 via oxidative phosphorylation. Lactate can also be used as an alternative energy source and facilitates rapid cell growth [10]. The Warburg effect in hematological or solid malignancy is well described in the literature [8, 11]. Most of the cases are associated with lymphomas and acute leukemias, but multiple myelomas are also reported [12, 13, 14, 15, 16]. Moreover, it has been hypothesized that cancer cells induce aerobic glycolysis on stromal cells, that in turn produce lactate and pyruvate, re‐incorporated by the cancer cells for the Krebs cycle and oxidative phosphorylation. This is known as the “reverse Warburg effect” [9, 17], and has specifically been described in myeloma cells [18].

Increased glycolysis also explains an often coexisting hypoglycemia with the Warburg effect [19, 20, 21, 22]. Hypoglycemia can also result from intense glucose consumption by an important tumor cell mass [8, 19, 23] or from liver tumor infiltration. Other known mechanisms include the production of insulin‐like substance by malignant cells and auto‐antibodies to insulin receptors, called insulin auto‐immune syndrome. Myeloma cells have been shown to possess functional insulin and IGF1 receptors [24, 25]. The literature reports rare cases of monoclonal gammapathy of undetermined significance (MGUS) and multiple myeloma associated with the presence of anti‐insulin antibodies, causing hypoglycemia. Auto‐immune hypoglycemia disappears under chemotherapy and reappears at relapse [26, 27]. Despite persistent hypoglycemia, our patient did not present any neuroglycopenic symptoms. This fact has already been described [23] and is explained by the use of lactate as a major alternative metabolic source for the brain. In our patient, hypoglycemia could be multifactorial, but its evolution is well correlated with the serum lactate level (Figure 1), suggesting a relationship with the myeloma cell mass.

However, in a critically ill patient presenting a hematological malignancy with lactic acidosis and hypoglycemia, other diagnoses must be ruled out, before concluding to the Warburg effect. Our patient presented upon admission to the ICU a hypoxemic pneumonia with septic shock, which could explain lactic acidosis. However, despite hemodynamic and respiratory stabilization, serum lactate levels remained over 10 mmol/L. There was no evidence for mesenteric ischemia, no liver failure, no drugs inducing acidosis, and the patient was supplied with thiamine. Acute renal failure could not entirely explain lactic acidosis, as lactate level remained over 5 mmol/L despite continuous renal replacement therapy. We observed a correlation between the evolution of the myeloma, serum lactate level, and hypoglycemia, with a decreasing of the lactate level and normalization of glycemia after the administration of corticosteroids and chemotherapy, and a rise of serum lactate level and hypoglycemia at myeloma's relapse (Figure 1). This makes our case consistent with a tumor‐associated aerobic glycolytic lactic acidosis.

Interestingly, to the best of our knowledge, this case is the first description of a newly diagnosed myeloma immediately associated with type B lactic acidosis, as all other cases reported myelomas evolving for several years, with lactic acidosis appearing only in relapses (Table 1). This could be linked with the important myeloma cell mass presented by our patient, as witnessed by the massive bone marrow infiltration. The mortality rate of the Warburg effect is very high. Indeed, all reported cases of myeloma with type B lactic acidosis, including ours, died within 1 year (Table 1). However, understanding the importance of the metabolic shift to increased glycolysis operating in cancer cells has been a major step in the development of glycolysis inhibitors in solid cancers and is studied as a novel approach in the treatment of hematological malignancies [28].

**TABLE 1:** Summary of reported cases of myeloma associated with lactic acidosis

Case reference	Age	Gender	Diagnosis	Evolution since initial diagnosis	Serum lactate level at admission (mmol/L)	Hypoglycemia	Survival after onset of hyperlactatemia
[12]	58	Male	Kappa light chain	14 years	14.7	Not specified	2 weeks
[13]	60	Female	Not specified	3 years	8.5	No	Several weeks
[14]	55	Male	IgG kappa	5 years	6.3	Not specified	11 months
[15]	64	Female	IgG lambda	1 year	8.8	Yes	23 days
[16]	58	Male	Not specified	15 years	14.7	Not specified	14 days

Aerobic glycolytic lactic acidosis and hypoglycemia are serious metabolic abnormalities encountered in hematological malignancies, resulting from changes in the metabolic pathway of the malignant cells. When associated with multiple myeloma, this condition is correlated with the disease progression and has a very high mortality rate.

## AUTHOR CONTRIBUTIONS

All authors were involved in the management of the patient. Caroline Ziegler, Lev Volkov, and Agnès Melone collected the data. Caroline Ziegler, Lev Volkov, Aurore Perrot, and Sébastien Gibot wrote the manuscript.

## CONFLICT OF INTEREST

The authors declare that there is no conflict of interest that could be perceived as prejudicing the impartiality of the research reported.
